# Analysis of Individual Variations in Autonomic Responses to Urban and Forest Environments

**DOI:** 10.1155/2015/671094

**Published:** 2015-10-05

**Authors:** Hiromitsu Kobayashi, Chorong Song, Harumi Ikei, Takahide Kagawa, Yoshifumi Miyazaki

**Affiliations:** ^1^Ishikawa Prefectural Nursing University, 1-1 Gakuendai, Kahoku, Ishikawa 929-1210, Japan; ^2^Center for Environment, Health and Field Sciences, Chiba University, 6-2-1 Kashiwa-no-ha, Kashiwa-shi, Chiba 277-0882, Japan; ^3^Forestry and Forest Products Research Institute, 1 Matsunosato, Tsukuba, Ibaraki 305-8687, Japan

## Abstract

Autonomic responses to urban and forest environments were studied in 625 young male subjects. The experimental sites were 57 forests and 57 urban areas across Japan. The subjects viewed the landscape (forest or urban environment) for a period of 15 min while sitting on a chair. During this period, heart rate variability (HRV) was monitored continuously. The results were presented as histograms and analyzed with special reference to individual variations. Approximately 80% of the subjects showed an increase in the parasympathetic indicator of HRV (lnHF), whereas the remaining subjects showed a decrease in the parasympathetic activity. Similarly, 64.0% of the subjects exhibited decreases in the sympathetic indicator of HRV (ln[LF/HF]), whereas the remaining subjects showed opposite responses. Analysis of the distribution of HRV indices (lnHF and ln[LF/HF]) demonstrated the effect of forest environments on autonomic activity more specifically than the conventional analysis based on the difference in mean values.

## 1. Introduction

Recently, there has been growing interest in the effects of the natural environment on human health. Beneficial effects may include stress relief, improved cognition and physical activity, better social cohesion, and promotion of overall health and mental well-being [1]. The predisposition of humans to responding positively to the natural environment may be a result of past adaptation to natural environments for survival or ongoing well-being during evolution [2].

The psychological effects of exposure to a forest environment on emotions have been demonstrated by various researchers. Bowler et al. [3] performed a meta-analysis of the results of several studies on the effect of natural environments and concluded that the natural environment has a consistent effect of reducing negative emotions (anger, fatigue, or sadness). In addition, exposure to a forest environment may have a positive effect on psychiatric impairments, such as alcoholic depression [4].

In recent years, along with psychological responses, physiological responses to a forest environment have been investigated. Studies have demonstrated that exposure to a forest environment results in reduced physiological indicators for stress. For example, lower fluctuation in skin conductance, shorter pulse-transit time (suggesting lower blood pressure), lower tension in frontalis muscles, and lower heart rate were observed during exposure to a video of natural settings [2]. Exposure to real forest environments decreased salivary cortisol concentration [5, 6] and cerebral blood flow (indicating a relaxation in brain activity) [6] and increased natural killer (NK) cells (indicating an enhancement of the immune system) [7–9]. Furthermore, Ohtsuka et al. [10] reported that a long-term experience in forest environment has significantly reduced blood glucose levels in patients with diabetes.

The current study investigated the effects of forest environments on autonomic nervous activity using heart rate variability (HRV) as an indicator. The relationship between HRV and autonomic functions has been established by previous studies [11–13]. Use of HRV as a physiological indicator of stress is also well established. In addition, during recent years within the field of alternative medicine, the effect of acupuncture has been evaluated by HRV [14, 15].

Our previous studies have demonstrated an increase in the high frequency (HF) component and/or a decrease in the low frequency (LF)/HF ratio of HRV in forest environments [16–19]. Similar results have been observed in parks in urban areas [20, 21] or during exposure to indoor plants [22, 23]. The HF component of HRV is considered a marker of parasympathetic activity, whereas the LF component or LF/HF ratio is considered a marker of sympathetic activity [24]. Thus, the results of the abovementioned studies suggested a relative activation of the parasympathetic function.

Use of HRV for evaluating stress provides some advantages over alternative physiological measurements. HRV can be recorded continuously in a noninvasive manner. Furthermore, use of a portable heart rate monitor can provide ambulatory recording of HRV. These advantages might be maximized in field studies rather than experimental studies. Thus, HRV can be the appropriate indicator of physiological responses to forest environments.

One of the features of the current study is an analysis with special reference to the distribution characteristics of individual variations in the HRV response. Most previous studies on physiological responses to environments have focused on the differences in the mean value; individual variations were considered an error or impurities. Individual variations have been an underutilized resource in various fields of life sciences. Bennett [25] described the tendency of focusing on means as the “tyranny of the golden mean.” From the viewpoint of adaptation, individual variations should have biological and/or evolutionary significance and should decidedly not be viewed as an error or impurity. The mean value has certain significance as one of the representative values of a population; however, it represents no more significance than any other aspect of the physiological responses of a population.

Analysis of the physiological response focusing on individual variations may be challenging compared with an analysis focusing on the mean value because the analysis of individual variations requires a larger sample size. The current study investigated the frequency components of HRV in 625 young Japanese males in urban and forest environments. The current study utilized a large sample size and can therefore provide a new perspective on the physiological responses to natural environments.

## 2. Materials and Methods

### 2.1. Study Sites and Subjects

The study sites were 57 forest and 57 urban areas across Japan. The chosen urban sites were downtown or nearby a Japan Railway (JR) station.

Although 684 young Japanese male university students participated in the experiments, only 625 subjects with complete data for both urban and forest sites were included in the analysis. The demographic parameters of the subjects are shown in Table 1.

None of the subjects reported a history of physical or psychiatric disorders. Consumption of alcohol and tobacco was prohibited and consumption of caffeine was controlled during the study period. The study was performed according to the regulations of the Ethics Committee of the Center for Environment, Health, and Field Sciences, Chiba University, or the Institutional Ethics Committee of the Forestry and Forest Products Research Institute in Japan.

### 2.2. Physiological Measurements

HRV was measured using a portable electrocardiograph (Activtracer AC-301A, GMS, Japan). Spectral analysis of HRV was conducted for 15 min recordings using HRV software (MemCalc/Win, GMS, Tokyo, Japan) based on the maximum entropy method. The HF and LF components were obtained by integration of the power spectra at the respective ranges of 0.15–0.40 and 0.04–0.15 Hz. The natural logarithms of the HRV indices (lnHF, ln[LF/HF]) were then calculated, considering that the raw HRV components indicate skewed distributions [26]. In the current study, HRV was measured during spontaneous breathing, and paced breathing was not applied. The subjects were instructed to avoid irregular breathing during the measurements. A previous study has reported that paced breathing has a negligible effect on interindividual variations in the spectral components of HRV [27].

### 2.3. Experimental Design

The experiment was performed at each experimental site over 2 consecutive days. Before the experiment, the aim of the study and the experimental protocol was explained and general instructions were provided to the subjects. The subjects ate lunch between 11:30 and 12:30, and the measurements were conducted between 13:30 and 15:30. All subjects were nonsmokers. Alcohol intake and unusual physical activity on the day before the measurement were forbidden.

The subjects at each site were randomly divided into two groups, and the order of exposure to the experimental conditions (urban or forest) differed among the two groups. One group was exposed to the forest site prior to the urban site, and the other group followed the reverse order. All subjects remained in a waiting room before moving to the field site. All subjects were instructed to rest on a chair for 5 min, which mitigated the physiological effects of any possible physical activity before the measurement period. HRV measurements were obtained during 15 min when the subjects viewed the landscape. On the second day, the subjects switched the field sites. The experimental protocol on the second day was the same as that on the first day.

Among the experiments at 57 sites, the experimental design used at 44 sites was the “Stay-in Forest Therapy” design, where arrangements were made for all subjects to reside in a hotel with identical single rooms. At the remaining 13 sites, the experimental design of “One-Day Forest Therapy” was used, where the subjects were allowed to return home after the first day. To reduce inconvenience to subjects and to limit research expenses, we switched to the simplified experimental design of One-Day Forest Therapy.

### 2.4. Data Analysis

Mean, median, standard deviation (SD), 5th and 95th percentile values, skewness, and kurtosis were calculated for each HRV index. Skewness is a measure of symmetry of distribution. Negative or positive skewness is indicated when the left or right tail of the research data fitted to a histogram is longer, respectively. The skewness of a normal distribution is zero. Kurtosis is a measure of whether the distribution curve is peaked (positive) or flat (negative) relative to the normal distribution. The kurtosis of a normally distributed data set is zero. Statistical analysis was performed using IBM SPSS statistics ver. 21 (IBM, New York, US).

## 3. Results and Discussion

### 3.1. Individual Variations in Autonomic Responses to a Forest Environment

**Figure 1:**
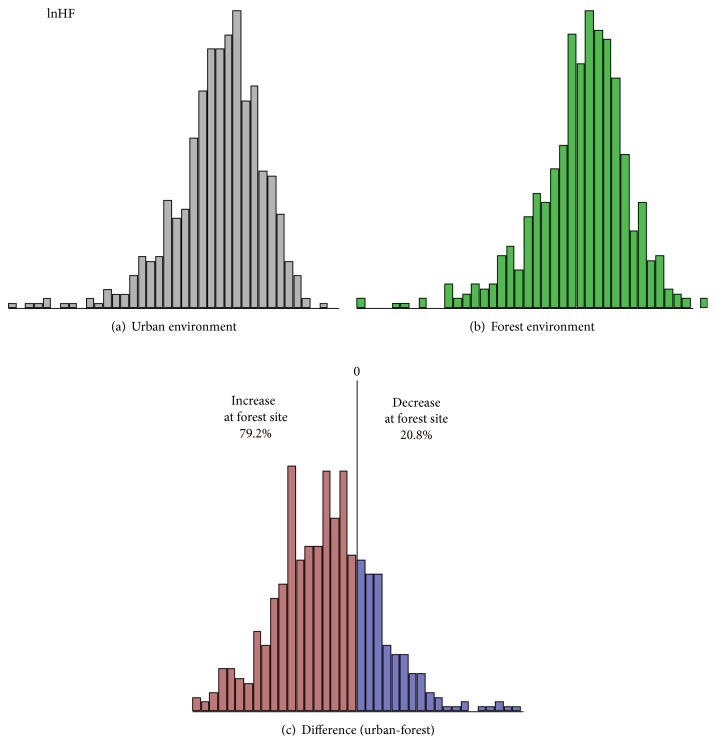
Histograms showing the high frequency component (lnHF) of heart rate variability in urban and forest environments. (a) lnHF at urban sites, (b) lnHF at forest sites, and (c) difference in lnHF between urban and forest sites.

The descriptive statistics of the distribution of the HRV indices are summarized in Table 2. The mean lnHF in urban and forest environments was 5.54 and 6.02 [ln(ms2)], respectively. A larger lnHF value was observed in forest environments, suggesting activation of the parasympathetic function. The histograms for lnHF are shown in Figure 1. Although the mean values were different, the distribution curves were almost identical between urban and forest environments. In both environments, the distributions indicated slightly left skewed and peaked curves compared with the normal distribution (Figures 1(a) and 1(b)). Differences in lnHF between urban and forest environments were calculated individually and depicted as a histogram (Figure 1(c)). Negative values in the difference imply that lnHF was larger in forest than in urban environments, whereas the positive values in the difference imply that lnHF was smaller in forest than in urban environments. A histogram showing the difference indicated a slightly left-skewed and markedly peaked distribution. In the results of the current study, 495 out of 625 (79.2%) subjects exhibited an increase in lnHF in forest environments.

**Figure 2:**
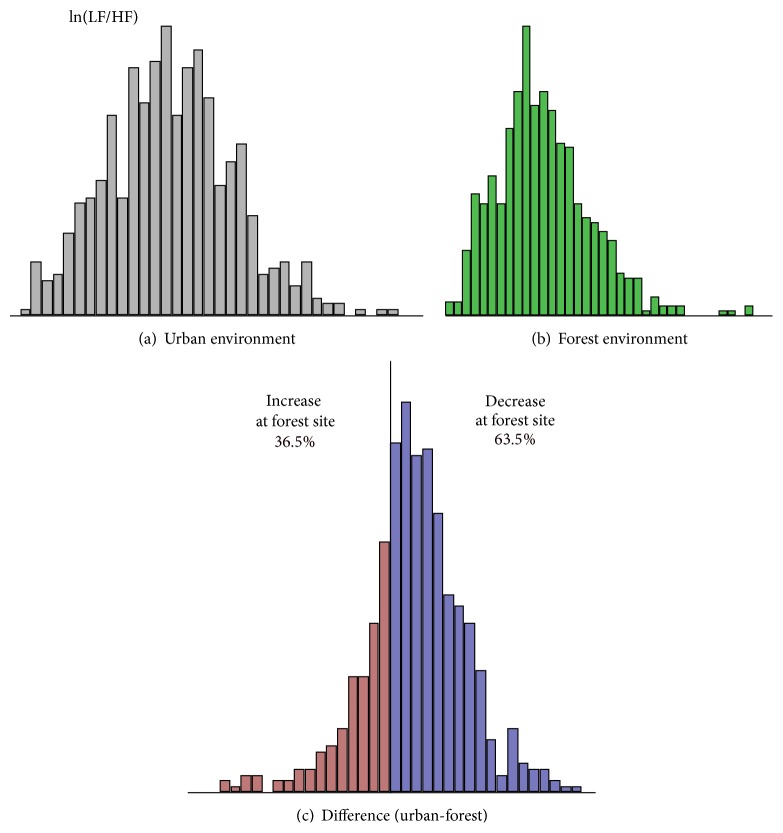
Histograms showing the low frequency/high frequency ratio (ln[LF/HF]) of heart rate variability in urban and forest environments. (a) ln(LF/HF) at urban sites, (b) ln(LF/HF) at forest sites, and (c) difference in ln(LF/HF) between urban and forest sites.

The results of ln(LF/HF) were analyzed in a similar way to lnHF. The mean ln(LF/HF) in urban and forest environments was 1.48 and 1.32, respectively. Lower ln(LF/HF) values were observed in forest environments, suggesting suppression of the sympathetic function. The histograms for ln(LF/HF) are shown in Figure 2. A histogram for the difference in ln(LF/HF) between urban and forest environments indicated an almost symmetrical distribution (Figures 2(a) and 2(b)). Furthermore, 397 out of 625 (63.5%) subjects exhibited a decrease in ln(LF/HF) in forest environments (Figure 2(c)).

In summary, approximately 80% of the subjects showed an increase in the parasympathetic activity in forest environments, whereas the remaining subjects exhibited a negative effect of the forest environments. The mean lnHF in urban and forest environments was 5.54 and 6.02 [ln(ms2)], respectively; thus, the change was approximately 9%. Although the difference in the mean values was statistically significant, the difference appeared marginal. On the other hand, the ratio of positive and negative responders (80 : 20) was a more conclusive result as compared with the changes in the mean values. Similar results were obtained for the sympathetic indicator [ln(LF/HF)]. Thus, histograms are considered to be an efficient tool for the analysis of physiological responses to natural environments in which larger individual variation exists.

### 3.2. Effect of Respiration and Air Pollution

It has been known that HRV is closely related with respiratory rate. Slower respiratory rate produces larger HF power in the HRV spectrum. Increased HF in a forest environment may relate with slower and/or deeper respiration. Gladwell et al. [28] studied the effects of natural and urban landscapes on heart rate, blood pressure, and respiration. In their results, no significant changes were observed in the respiratory rate and depth, although a parasympathetic indicator of HRV increased in natural landscape similar to the present results. Thus, the change in the respiratory rate is not considered to be a major cause of the increased HF in forest.

In recent years, the effect of air pollution on human HRV has been attracting attention in the field of environmental medicine. A relationship between increased PM2.5 (particulate matter with an aerodynamic diameter of <2.5 *μ*m) and decreased parasympathetic indicator of HRV has been reported [29, 30]. Because the effect of PM2.5 was considered to be acute rather than chronic [31], the difference in air pollution between urban and forest environments may be an explanation of the present results.

### 3.3. Biophilia and Biophobia

The biophilia hypothesis was proposed by the distinguished biologist Wilson [32]. Biophilia is defined as the “innate tendency to focus on life and life-like processes” [33]. This tendency could be explained from an evolutionary perspective. For millions of years, human beings (or their recent ancestors) lived in the savannas of Africa. Within this environment, natural features such as trees or forests could provide food, water, or shelter, thereby increasing the probability of survival. Thus, biophilia can be regarded as an adaptive characteristic of human evolution.

On the other hand, it has been known that certain people show a strong dislike for natural settings. This tendency is called biophobia [34]. Biophobia includes certain specific phobias, such as arachnophobia (irrational fear of spiders) or entomophobia (fear of insects). Previous studies have reported that patients displaying arachnophobia showed increased HR or HRV during presentation of images of spiders [35, 36]. Without actually perceiving spiders or insects, people exhibiting these phobias manifested phobic reactions even if they merely imagined spiders or insects in their immediate environment. Therefore, the negative responders in the results of the current study could be explained by these specific phobias to living things. Further consideration of the relationship between physiological responses to the forest environment and biophobia is expected in future studies.

## 4. Conclusion

Autonomic responses to a forest environment were studied in 625 young male subjects. The results were demonstrated using histograms and were analyzed with special reference to individual variations. An increase in the parasympathetic indicator of HRV (lnHF) was observed in approximately 80% of the subjects. Analysis of the distribution of HRV indices (lnHF and ln[LF/HF]) demonstrated the effect of forest environments on autonomic activity more specifically than the conventional analysis based on mean values.

## Figures and Tables

**Table 1:** Demographic parameters of the subjects (*n* = 625).

	Age (years)	Height (cm)	Weight (kg)
Mean	21.6	172.3	64.7
SD	1.6	5.6	9.6
Max	29	188	110
Min	19	155	42

^*∗*^SD: standard deviation.

**Table 2:** Descriptive statistics of the distribution of heart rate variability (*n* = 625).

	ln⁡HF			ln⁡(LF/HF)		
	Urban	Forest	Urban-forest	Urban	Forest	Urban-forest
Mean	5.54	6.02	−0.48	1.48	1.32	0.16
Median	5.66	6.14	−0.45	1.51	1.31	0.16
SD	0.92	1.01	0.70	0.77	0.82	0.71
5th percentile	3.81	4.41	−1.57	0.16	0.00	−0.92
95th percentile	6.9	7.29	0.56	2.70	2.66	1.19
Skewness	−1.20	−1.10	−0.92	−0.21	0.32	−0.46
Kurtosis	3.42	2.97	8.21	0.47	0.97	4.24

^*∗*^SD: standard deviation; skewness: a measure of symmetry of distribution; Kurtosis: a measure of whether the distribution curve is peaked (positive) or flat (negative) relative to the normal distribution.
